# Perinatal effects of maternal FT3/FT4 ratio on gestational transient thyrotoxicosis

**DOI:** 10.20945/2359-3997000000371

**Published:** 2021-07-16

**Authors:** Eren Gürkan, Kenan Dolapçıoğlu, Emre Dirican

**Affiliations:** 1 University of Mustafa Kemal Department of Endocrinology and Metabolism Hatay Turkey Department of Endocrinology and Metabolism, University of Mustafa Kemal, Hatay, Turkey.; 2 University of Mustafa Kemal Department of Obstetrics and Gynecology Hatay Turkey Department of Obstetrics and Gynecology, University of Mustafa Kemal, Hatay, Turkey.; 3 University of Mustafa Kemal Department of Medical Informatics and Biostatistics Hatay Turkey Department of Medical Informatics and Biostatistics, University of Mustafa Kemal, Hatay, Turkey.

**Keywords:** Gestational transient thyrotoxicosis, maternal weight, birth weight, gestational age, thyroid function tests

## Abstract

**Objective:**

The effects of maternal thyroid hormone levels on the course of pregnancy and birth weight have attracted interest. The aim of the present study was to consider FT3 and FT3/FT4 ratio in the evaluation of the effects of maternal thyroid functions in gestational transient thyrotoxicosis (GTT).

**Materials and methods:**

This case-control study included 45 patients with GTT and 45 healthy pregnant women. Maternal history before pregnancy, thyroid function tests, thyroid autoantibodies, and thyroid ultrasonography results in 6th to 10th weeks of pregnancy were used in the differential diagnosis of GTT. In both groups, the effects of FT3, FT4 and FT3/FT4 ratios on gestational age and birth weight were evaluated.

**Results:**

There was no significant difference in the gestational age between the GTT and control groups (39,3±1,0 weeks and 39,2±1,2 weeks, respectively). Birth weights were similar in both groups (3205,2±4899 g and 3196,6±309,3 g, respectively). When maternal weight was adjusted, a positive correlation was observed between maternal FT3/FT4 ratio and birth weight (r=0,317, p=0,017). Additionally there was a positive correlation between the gestational age and the birth weight in the control group (ρ=0,726, p=0,001).

**Conclusion:**

GTT had no significant effect on the gestational age and the birth weight. On the other hand an increase in the maternal FT3/FT4 ratio had a positive effect on the birth weight in the patient with GTT. Maternal characteristics (age, weight, BMI) and FT3/FT4 ratio should be taken into consideration in future impact assessment studies on this issue.

## INTRODUCTION

Mild hyperthyroidism may occur in early stages of pregnancy due to an effect of increased human chorionic gonadotropin hormone. This condition is defined as gestational transient thyrotoxicosis and it is the most common cause of thyrotoxicosis in the first trimester of pregnancy. The incidence of GTT during pregnancy varies between 1-3%. While serum thyroid-stimulating hormone (TSH) levels are suppressed in GTT, FT4 and FT3 levels are normal or increase. The most important serological characteristic of patients with overt GTT is high FT3 level ( 1 – 4 ).

Gestational transient thyrotoxicosis is distinguished from Graves’ disease by absence of hyperthyroidism before pregnancy, absence of ophthalmopathy and goiter on physical examination, lack of TSH receptor antibody in serum, and normalization of thyroid functions in the second trimester ( 4 ). There is no indication for using antithyroid drugs in GTT ( 5 ).

Presence of hyperthyroidism during pregnancy is associated with low birth weight of a newborn. Compared to patients without hyperthyroidism during pregnancy, the rate of low birth weight increases by 2.3 times in patients with controlled hyperthyroidism and 9.2 times in those with uncontrolled course. This situation can be often associated with premature birth ( 6 ). In the study by Lazarus and Kaklamanou ( 7 ), higher rate of poor pregnancy outcomes were observed in the pregnant women with Graves’ disease compared to those with GTT.

In pregnant women with hyperthyroidism who achieve euthyroid state with treatment, the risk of reduced fetal growth, premature birth, and low birth weight increase. In addition, pregnancy-induced hypertension can also be at a high rate in these women ( 8 ). Maternal FT4 levels in the early period of pregnancy and its perinatal effects have also been investigated ( 9 ). The aim of the present study was to consider FT3 and FT3/FT4 ratio in the evaluation of the effects of maternal thyroid functions in GTT.

## MATERIALS AND METHODS

The present study was designed as a retrospective case-control study in which data of patients were obtained through the hospital records. The present study was approved by the Ethics Committee of Hatay Mustafa Kemal University (date: 27.02.2020 and approval no: 03).

Data of 45 patients who applied to the endocrinology outpatient clinic of Hatay Mustafa Kemal University Faculty of Medicine from September 2015 through December 2016 and who were diagnosed with thyrotoxicosis via thyroid function tests (TFTs) between the 6^th^ and 10^th^ weeks of their pregnancies were analyzed. Absence of a known thyroid disease, serum TSH levels being <0.1 IU/mL, normal or high serum FT4 and/or FT3 levels, and negative thyroid auto-antibodies (anti-TPO, anti-Tg, and TSH receptor antibody) were accepted as the basic criteria for the diagnosis of GTT ( 10 ). Patients who previously received radiotherapy, had thyroid disease before pregnancy, and had a history of gestational trophoblastic disease were excluded. Patients losing 5% of their body weight during pregnancy or having ketonuria and frequent vomiting were considered to have hyperemesis gravidarum and they were also excluded from the study. Control group was composed of 45 pregnant women who applied to the gynecology and obstetrics outpatient clinic within the same dates and who were between the 6^th^ and 10^th^ weeks of their pregnancies. The women in the control group were followed-up by the same physician, had normal TFT (FT3, FT4, and TSH) values and normal thyroid ultrasonography findings, had negative thyroid autoantibodies, and had no additional systematic diseases.

On the patients’ monitoring system, the patients with GTT had monthly follow-up records until their TFT values were normalized in 2^nd^ trimester. The control group had at least one normal TFT result in each trimester until the end of their pregnancy periods. Data relating to the pregnancy and perinatal outcomes were obtained from both the maternity service records and the pregnant follow-up system in our city. Besides, missing data in the anamnesis of the patients were obtained by phone calls to the patients.

In our clinic, blood samples were routinely collected in the morning after overnight fasting. For TFT and thyroid autoantibodies, chemiluminescent immunoassay technique (Cobas E6000, Roche Diagnostic, Germany) was used. Ultrasonographic measurements were performed by an experienced radiologist using the MyLab ClassC ultrasonography device (Esaote SpA, Italy) with a 14-MHz linear prob.

### Statistical analysis

Data were analyzed using the Statistical Package for the Social Sciences for Windows, Version 21.0 (IBM Corp., Armonk, NY, USA). In descriptive statistics, numerical variables were expressed as mean, standard deviation, median, minimum, and maximum and categorical variables were expressed as number and percentage. For the normality analysis of variables, the Shapiro-Wilk test was used. In the analyses, student-t test, Mann-Whitney U test, Chi-square test, Pearson correlation, Spearman's rank correlation, and Partial correlation were used. While drawing scatter graphics in partial correlation, unstandardized residual was calculated and drawn over linear regression. A p value <0.05 was accepted as a statistical significance limit within 95% confidence interval.

## RESULTS

There were no significant differences in maternal characteristics (age, weight, and BMI) between the GTT and control groups ( Table 1 ). The median number of births was slightly higher in the GTT group than in the control group, but the difference was borderline significant (2.5 vs. 2, p=0.053). As expected, FT4, FT3, and TSH values were statistically significantly higher in the GTT group as compared to controls (p=0.001 for all). There were no significant differences between the GTT and control groups according to the gestational ages during birth, birth weights, and sex of the newborns (p=0.882, p=0.918, and p=0.527, respectively) ( Table 1 ).

**Table 1:** Characteristic data of our study groups

Characteristics		GTT group		Control group		p
		Mean ± SD	Median (min-max)	Mean ± SD	Median (min-max)	
Age (year)		30.1 ± 6.4	30 (18-44)	32.2 ± 5.7	32 (20-49)	0.075 a
Weight (kg)		66.5 ± 13.1	68 (50-98)	64.8 ± 9.5	63.5 (49-87)	0.718 b
BMI (kg/m^2^)		25.4 ± 4.7	24.9 (18.8-37.5)	24.8 ± 4.5	23.8 (19.5-39.7)	0.505 b
Number of births		2.7 ± 1.6	2.5 (1-7)	1.9 ± 0.8	2 (1-4)	0.053 b
FT4 (ng/dL)		1.9 ± 0.7	1.75 (0.9-3.8)	1.2 ± 0.2	1.2 (0.5-1.7)	0.001 b
FT3 (pg/mL)		5.0 ± 1.4	4.9 (2.7-9.0)	3.2 ± 1.1	3.2 (1.2-5.5)	0.001 b
FT3/FT4		2.8 ± 0.5	2.7 (1.5–3.8)	2.8 ± 1.1	2.5 (1.1–6.0)	0.215 b
TSH (IU/mL)		0.02 ± 0.02	0.01 (0.00-0.01)	1.72 ± 0.61	1.70 (0.30-3.26)	0.001 b
Gestational age during birth (wk)		39.3 ± 1.0	40 (37-40)	39.2 ± 1.2	40 (37-41)	0.882 b
Birth weight (g)		3205 ± 490	3245 (1480-4300)	3197 ± 309	3240 (2640-3930)	0.918 a
Newborn sex (n (%))						
	Girl	25 (53.2)		22 (46.8)		0.527 c
	Boy	20 (46.5)		23 (53.5)		

a Student-t Test

b Mann-Whitney U Test

c Chi-Square Test, wk: week.

BMI: body mass index; GTT: gestational transient thyrotoxicosis; SD: standard deviation; TSH: thyroid-stimulating hormone.

**Figure 1:**
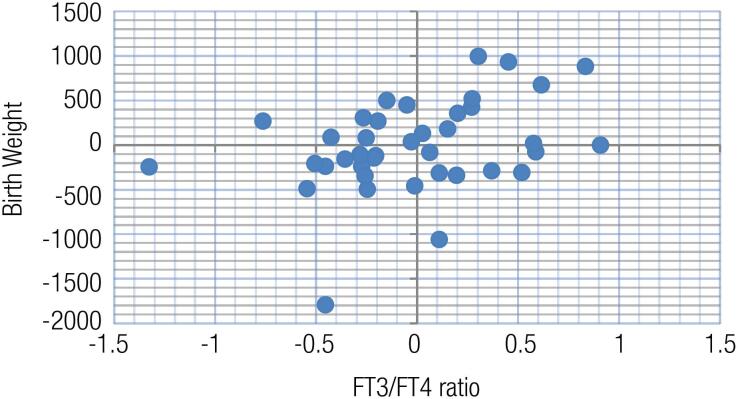
Partial correlation analysis between the birth weight and FT3/FT4 ratio in the gestational transient thyrotoxicosis group (weight control variable). Values in the graphic are calculated as per non-standardized residues.

**Figure 2:**
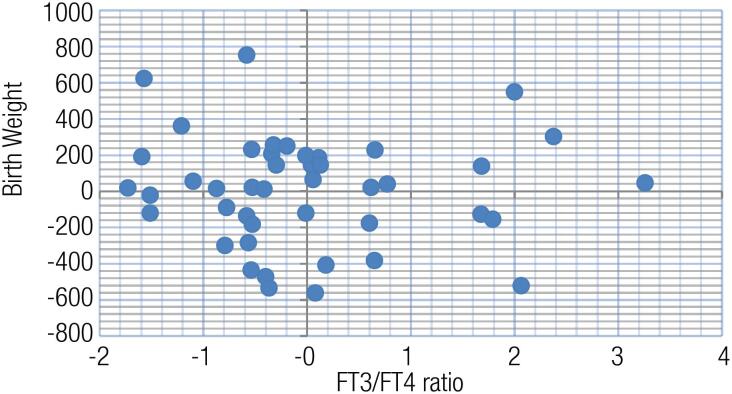
Partial correlation analysis between the birth weight and FT3/FT4 ratio in the control group (weight control variable). Values in the graphic are calculated as per non-standardized residues.

There was no relationship between the weight and birth weight (r=0.019, p=0.908) in the GTT group. When the effects of maternal thyroid hormones were evaluated, a positive correlation was found between the maternal weight and FT3/FT4 ratio (r=0.302, p=0.049) in this group. A positive correlation was observed between the birth weight and FT3/FT4 ratio in the GTT group (r=0.317, p=0.017) ( Table 2 and Figure 1 ). However, no such correlation was obtained in the control group ( Table 2 and Figure 2 ). On the other hand, when weight, as a confounding factor, was adjusted in the partial correlation analysis, there was no significant correlation between the FT3/FT4 ratio and the gestational week in both study groups (r=0.146 and p=0.376 in the GTT group and r=0.050 and p=0.752 in the control group).

**Table 2:** Correlation analysis between maternal thyroid hormones and birth weight by adjusting weight as a confounding factor in the study groups

	Confounding factor	Parameters	Statistics	Birth weight
GTT group	Weight	FT4	r	−0.093
			p	0.577
		FT3	r	0.131
			p	0.435
		FT3/FT4	r	0.317
			p	0.017
Control group		FT4	r	−0.012
			p	0.941
		FT3	r	−0.066
			p	0.677
		FT3/FT4	r	−0.067
			p	0.674

According to the Spearman's rank correlation analysis results in the study groups, the increase in the gestational age in the GTT group had no significant effect on the birth weight (p=0.194, p=0.140). However, such correlation was found significant in the control group (p=0.726, p=0.001).

## DISCUSSION

In the current case-control study including the patients with GTT and the pregnant women having normal thyroid functions, the mean gestational age of the groups during birth were compared and no significant difference was obtained (39.3±1.0 weeks in the GTT group and 39.2±1.2 weeks in the control group, p=0.882). Similarly Kinomoto-Kondo and cols. ( 11 ) evaluated the effects of GTT on pregnancy outcomes in their case-control study. While they reported no significant difference between the GTT patients and controls regarding low birth weight, preeclampsia, pregnancy-induced hypertension, ablation placenta, and prematurity, they found the gestational age at birth being significantly lower in the GTT group ( 11 ). In the present study, no negative effects of GTT on pregnancy-related results (preeclampsia, ablation placenta, prematurity, gestational diabetes, etc.) were observed too. However, the discrepancy observed in the results of gestational age at birth between the present study and the above-mentioned study could be attributed to the difference in the number of patients included in the studies.

According to the results of the correlation analysis between the birth weight and gestational age in the present study, the increase in the gestational age in the GTT group did not affect the birth weight (p=0.194 and p=0.140). However, a positive significant correlation was observed between the birth weight and gestational age in the control group (p=0.726 and p=0.001). This correlation observed in the control group may help to explain the difference in birth weights between our study and other studies. In our study, gestational age in patients with GTT was >37 weeks and this was compatible with the literature ( 11 ).

In the present study, as expected, maternal FT4 and FT3 values were significantly higher and maternal TSH values were significantly lower in the GTT group as compared to those in the control group. However, these parameters had no significant effect on gestational age and birth weight. In the study by Kinomoto-Kondo and cols. ( 11 ), a negative correlation was found between the first trimester maternal FT4 levels and gestational age.

In the studies evaluating the effects of thyroid functions on pregnancy, it has been reported that an increase in maternal FT4 level in pregnant women achieving euthyroid state or having GTT negatively affects gestational age and birth weight ( 11 – 13 ). However, in the present study, there was no correlation between the FT4 level and gestational age and birth weight. In the FaSTER study, the negative correlation between maternal FT4 levels and birth weight in pregnant women achieving euthyroid state was also confirmed ( 14 ). However, the effects of FT3 level and FT3/FT4 ratio on this analysis were not investigated; this was stated as the limitation of the study. It was also emphasized that maternal FT3/FT4 ratio was a stronger metabolic indicator than FT4 alone ( 14 ). In the present study, when the effect of FT3/FT4 ratio on birth weight and gestational age was evaluated, a positive correlation was found between the maternal FT3/FT4 ratio and birth weight in the GTT group. In the FaSTER study, FT4 level was negatively correlated with the maternal age and weight ( 14 ). Considering this situation, the correlations of FT4 and FT3/FT4 ratio with the maternal age and weight were also examined in the present study groups. A positive correlation was found between the FT3/FT4 ratio and the maternal weight in the group with GTT. To the best of our knowledge, there has been no analysis on this issue in patients with GTT in the literature. When the correlation analysis was performed considering the effect of maternal weight as a confounding factor, a positive correlation was obtained between the FT3/FT4 ratio and the birth weight in our GTT group. This result could help us to explain the normal birth weight in our patients with GTT. As one of the plasma thyroid hormone levels increases, the other decreases. FT3 is an active thyroid hormone and stimulates endogenous glucose production. This is related with relatively decreased FT4 level and increased maternal glucose production ( 15 ).

The major limitation of the present study is being a single center and small-scale study. On the other hand, in accordance with the literature, regular follow-up of the patients owing to our pregnancy tracking system is one of the advantageous of the present study.

In conclusion, no significant difference was found between the birth weight and gestational age when comparing the patients with GTT with the healthy controls. On the other hand, maternal FT3/FT4 ratio had a positive correlation with the birth weight in the GTT group. Maternal characteristics (age, weight, BMI, etc.) and FT3/FT4 ratio should also be taken into account when conducting an impact assessment study in patients with GTT. Future multi-center prospective studies are needed on this subject.
